# Highlighting recent achievements to advance more effective cancer immunotherapy

**DOI:** 10.1186/s13046-025-03316-8

**Published:** 2025-02-18

**Authors:** Beatrice Belmonte, Sheila Spada, Paola Allavena, Matteo Benelli, Vincenzo Bronte, Giulia Casorati, Lorenzo D’Ambrosio, Roberto Ferrara, Anna Mondino, Paola Nisticò, Roberta Sommaggio, Marcella Tazzari, Claudio Tripodo, Antonio Sica, Pier Francesco Ferrucci

**Affiliations:** 1 Tumor Immunology Unit, Departmentof Health Sciences, University of Palermo, Palermo, Italy; 2 Tumor Immunology and Immunotherapy Unit, IRCCS-Regina Elena National Cancer Institute, Rome, Italy; 3 IRCCS Humanitas Research Hospital, Milan, Italy; 4 Bioinformatics Unit, Hospital of Prato, Prato, Italy; 5 Veneto Institute of Oncology IOV-IRCCS, Padua, Italy; 6 Experimental Immunology Unit, DivisionofImmunology,TransplantationandInfectiousDiseases, IRCCS San Raffaele Scientific Institute, Milan, Italy; 7 Department of Oncology, University of Turin, Orbassano, TO Italy; 8 Department of Medical Oncology, ScientificInstituteforResearch,HospitalizationandHealthcare(IRCCS), San Raffaele Scientific Institute, Milan, Italy; 9 Lymphocyte Activation Unit, Division of Immunology, Transplantation and Infectious Diseases, IRCCS San Raffaele Scientific Institute, Milan, Italy; 10 Department of Surgery, Oncology and Gastroenterology, University of Padova, Padua, Italy; 11 Advanced Cellular Therapies and Rare Tumors Unit, IRCCS Istituto Romagnolo per lo Studio dei Tumori (IRST) “Dino Amadori” S.r.l., Meldola, Italy; 12 IFOM ETS, The AIRC Institute of Molecular Oncology, Milan, Italy; 13 Department of Oncology and Heamato-Oncology, University of Milan, Milan, Italy; 14 Department of Pharmaceutical Sciences, University of Piemonte Orientale “A. Avogadro”, Novara, Italy; 15 IRCCS Humanitas Research Hospital, Rozzano, Milan Italy; 16 Department of Oncology Gruppo Multimedica, Milan, Italy

## Abstract

From 17 to 19th October 2024, the XXI Italian Network for Bio-Immunotherapy of Tumors Meeting (NIBIT) took place in Palermo, in the marvelous historical location of Teatro Politeama, under the auspices of the Italian Association of Medical Oncology (AIOM), Italian Association of Cancer Research (AIRC), Fondazione Pezcoller, Italian Alliance against Cancer (ACC), Italian Lymphoma Foundation (FIL), Grazia Focacci Foundation and Melagioco Foundation. The conference covered a spectrum of topics ranging from target discovery to therapeutic advances in immuno-oncology, bringing world-renowned experts to present groundbreaking innovations in basic, translational, and clinical cancer research. Six sessions focused on cellular therapies, digital pathology, vaccines, tertiary lymphoid structures, and microenvironment in order to get deep insights on how to personalize diagnosis and therapies in the clinical setting. Young investigators had the opportunity to meet and greet their mentors, promoting the synergy of the academic and industrial sectors within the national and international panorama, discussing the application of artificial intelligence on multi-specific antibodies, drug conjugates, and antibody fusion proteins that are advancing the efficacy of precision medicine and minimizing off-target effects.

## Session 1

### Cellular therapies: present and future

Session 1 was co-chaired by Giulia Casorati (Milan, Italy) and Roberta Sommaggio (Padova, Italy) focused on recent advances in cellular therapies both on CAR-T in lymphomas and Tumor Infiltrating Lymphocytes (TILs) in melanoma, including clues on novel strategies targeting diverse CMV epitopes across various HLA types.

Anna Sureda (Barcelona, Spain) presented how cellular therapy strategies look like in lymphomas in 2024 and provided an in-depth overview of hematopoietic cell transplantation (HCT) and CAR-T cell therapies for lymphomas, emphasizing recent advancements and comparative outcomes. She discussed the treatment landscape for aggressive B-cell lymphomas such as diffuse large B-cell lymphoma (DLBCL), mantle cell lymphoma (MCL), and follicular lymphoma (FL), highlighting clinical trials and real-world evidence from registries (https://www.ebmt.org/).

She focused on a new treatment algorithm for relapsed or refractory (RR) DLBCL showing the importance of the time of relapsing after the first line of therapy, less or more than 1 year, demonstrating the efficacy of autologous CD19 CAR-T cells as a second-line treatment, with data supporting CAR-T cells' superiority in progression-free survival over standard-of-care HCT in high- risk cases [1]. For MCL, CAR-T therapy is increasingly favored over allogeneic HCT due to its curative potential with lower treatment-related mortality [2–4]. Lastly, the role of HCT in FL is noted to be diminishing with the emergence of effective CAR-T options, suggesting a paradigm shift in cellular therapy strategies for lymphomas [5].

The presentation "Prime Time for Adoptive T-Cell Therapy for Metastatic Melanoma" by Inge Marie Svane (Herlev, Denmark) highlighted the advancements in adoptive cell therapy (ACT) with tumor- infiltrating lymphocytes (TILs) as a treatment for metastatic melanoma, particularly for patients who are resistant to checkpoint inhibitors [6–8]. TIL therapy has shown promising outcomes in achieving durable responses, with a progression-free survival (PFS) benefit and a higher overall response rate (ORR) compared to traditional therapies, such as ipilimumab [9, 10]. Results from a phase III trial demonstrated significantly improved PFS and response rates for TIL therapy, underscoring its potential as a standard treatment option. Additionally, she explored innovative approaches to increase the efficacy of TILs in anti-PD1 resistant metastatic melanoma patients, including CRISPR-mediated PD-1 editing, IL-7-producing TILs (ADP-TILIL7), or reprograming tumor cells by genetic modification with cDC1 (NEO-TILs) to increase the antigen presentation. Future directions involve combining TIL therapy with oncolytic viruses and developing "armored" TILs to address resistance and improve clinical outcomes in metastatic melanoma [11, 12].

Cytomegalovirus (CMV) infections pose significant risks for immunocompromised patients, especially those undergoing hematopoietic stem cell transplantation (HSCT). While antiviral drugs like letermovir can suppress CMV reactivation during prophylaxis, late reactivations remain frequent due to delayed restoration of CMV-specific immunity. To address this, Elvira D’Ippolito (Munich, Germany) and her group proposed prophylactic transfer of HSCT donor T cells engineered with CMV-specific T-cell receptors (TCRs) to high-risk CMV-seropositive patients receiving stem cells from CMV-seronegative donors. Their approach involves building a library of highly functional CMV-specific TCRs from CMV-seropositive healthy donors, targeting diverse CMV epitopes across various HLA types. These TCRs are delivered into T cells using an in-house, CRISPR-Cas9-based method called orthotopic TCR replacement (OTR). OTR simultaneously knocks out the endogenous TCR α and β genes and inserts a transgenic TCR into the TCR α locus. This innovative method is being implemented into a GMP-compliant manufacturing process to enable clinical application [13, 14].

Oral Communications included presentations by Francesco De Sanctis, Elisa Cappuzzello and Gloria Delfanti.

Francesco De Sanctis (Verona, Italy) showed how the expression of the membrane tetraspanin Claudin 18, a protein found in tight junctions on cancer cells, promotes T Lymphocyte infiltration and antitumor immunity, addressing immunological challenges in pancreatic cancer, which is typically resistant to immunotherapy and characterized by low survival rates. He reported that the CLDN18 facilitates cytotoxic T lymphocyte (CTL) recruitment and activation in experimental models, suggesting its potential as a biomarker and therapeutic target. In patients with pancreatic ductal adenocarcinoma (PDAC), CLDN18 expression correlates with improved clinical outcomes and synergizes with TIL presence to predict survival. The findings underscore the significance of CLDN18 in enhancing immune surveillance in pancreatic cancer, paving the way for personalized therapeutic strategies leveraging CLDN18-driven immunomodulation [15].

Elisa Cappuzzello (Padova, Italy) presented innovative findings on the potential of Cytokine- Induced Killer (CIK) cells as an alternative to CD19 CAR T-cell therapy for treating aggressive B- cell malignancies resistant to chemo-immunotherapy [16]. CIK cells, which express the CD16 receptor (FcγRIIIa), and can mediate antibody-dependent cell cytotoxicity (ADCC), making them particularly suited for combination with monoclonal antibodies [17]. Cappuzzello's study evaluated the efficacy of combining CIK cells with anti-CD19/CD20 antibodies (Tafasitamab or Obinutuzumab) or the bispecific Blinatumomab (CD19xCD3) and compared it with CD19 CAR T-cell therapy in both in vitro and in vivo models. The results demonstrated that CIK cell combinations achieved similar tumor control to CAR T-cells but with reduced pro-inflammatory cytokine release, indicating a lower risk of cytokine release syndrome (CRS). This approach harnesses the cytotoxic potential of CIK cells without the need for genetic modification, providing a cost-effective and potentially safer alternative to CAR T-cell therapy, with promising implications for clinical application [18].

Gloria Delfanti (Milan, Italy) presented compelling findings on the role of CD1d-restricted invariant natural killer T (iNKT) cells in immunotherapy, particularly in the context of colorectal cancer (CRC) liver metastases. iNKT cells demonstrate a therapeutic advantage by modulating the tumor microenvironment (TME) to reduce pro-tumor myeloid populations and enhance immune activation. Delfanti’s study focused on iNKT cells engineered with a carcinoembryonic antigen-specific chimeric antigen receptor (CEA-CAR iNKT), which showed markedly improved tumor control over conventional T cells, achieving significant eradication of CRC liver metastases in treated mice. This proof-of-concept research establishes CEA-CAR iNKT cells as a promising therapeutic strategy, with data supporting their efficacy and safety as a novel approach to managing CRC liver metastases, setting a foundation for future clinical applications [19].

## SESSION 2

### Organoids and tissue fragments at the forefront of personalized oncology

Session 2, co-chaired by Antonio Sica (Milan, Italy) and Marcella Tazzari (Meldola, Italy), discussed recent advances in the use of Patient-Derived Organoids (PDOs) and Patient-Derived Explants (PDEs) in oncology precision medicine.

Salvatore Piscuoglio (Milan, Italy) and Giovanni Blandino (Rome, Italy) both discussed how the use of PDOs and PDEs will enhance therapeutic decision-making, emphasizing that the success of such an approach will largely depend on the ability of these systems to faithfully replicate not only the molecular phenotype of patients’ tumors but also the immune metabolic profile and micro physiology of the tumor microenvironment (TME). They further highlighted the importance of biobanking patient-derived tumor materials, such as peripheral blood mononuclear cells, tumor cells, and cancer-associated fibroblasts (CAFs), to create faithfully personalized and context-specific PDOs.

Salvatore Piscuoglio opened the session by discussing some limitations of PDOs. While complex in their multicellularity, they remain an in vitro model lacking certain features of in vivo tumors, particularly aspects of the TME. Piscuoglio also highlighted the advantages of using PDEs for drug activity screening. Nonetheless, he emphasized the primary strengths of PDOs, especially their high fidelity in replicating the genetic makeup of the original tumor, which allows for the optimization of therapies based on each patient’s unique cancer profile [20, 21]. The PDO approach has shown a high success rate in creating living biobanks, providing models that closely mirror the phenotype and therapeutic response of the tumors they derive from. To more faithfully replicate the TME, advancements in PDOs now include co-cultures with fibroblasts and immune cells, enabling studies on how cellular interactions influence the extracellular matrix (ECM) and immune-mediated killing mechanisms. Despite the advantages of 3D models, current limitations of this technology include the long growth time and still low predictive value for certain tumor types. These challenges suggest there is still significant progress to be made before PDOs can become fully reliable predictive tools for clinical decisions, particularly for investigating tumor-intrinsic and tumor-extrinsic resistance mechanisms.

Giovanni Blandino presented the workflow for PDOs in solid tumors, active at the Regina Elena

Institute. Blandino's laboratory has extensive experience in this field, with recent publications covering triple-negative breast cancer (TNBC) [22], advanced and metastatic colorectal cancer (CRC) [23] and non-small cell lung cancer (NSCLC) [24]. Using PDO models, they have demonstrated that genetic profiling, such as targeting the MYC-SPAG5 axis, can sensitize TNBC PDOs to cisplatin treatment [22] and explored drug repurposing strategies based on transcriptomic data [23]. In his presentation, Blandino advanced this work by highlighting his team’s success in validating PDOs for two additional cancer types: endometrial cancer (ENDC) and bladder cancer (BLC). The presented workflow suggests the benefits of a multidisciplinary approach, incorporating a wide array of technologies, such as high-throughput pharmacogenomic screening, to analyze PDO responses in real-time. According to Blandino, immunohistochemical validation and genomic concordance are essential prerequisites before evaluating PDO responses to standard-of-care drugs (SOCs) and, potentially, experimental therapies guided by genomic analyses. Blandino's team validated these PDOs, confirming their alignment with the original surgical tissue at the pathological, genomic, and transcriptomic levels. He was also successful in preserving immune cells within bladder cancer PDOs, confirming this achievement with bioinformatic deconvolution methods. Additionally, his group has characterized the integration and chemosensitivity of CAFs into endometrial cancer PDOs. The goal of Blandino's team is to incorporate these tumor miniatures into the Tumor Molecular Board portfolio (TMB), bridging the gap between laboratory models and personalized cancer treatment planning. Chiara Cattaneo (Milan, Italy) introduced an innovative approach using PDOs in personalized oncology. She presented a co-culture system of PDOs with T cells, enabling the induction of tumor- reactive T cells by pairing PDOs with autologous peripheral blood lymphocytes. This platform demonstrated its potential to facilitate the unbiased enrichment of tumor-reactive blood T cells from epithelial cancers, including mismatch repair-deficient colorectal cancer (CRC) and non-small cell lung cancer (NSCLC) patients. It also allowed for the assessment, at the individual patient level, of matched PDOs' sensitivity to T cell-mediated killing [25, 26].These tumor-specific T cells, enriched in an antigen-agnostic manner, could be a starting point for their ex vivo expansion for adoptive cell therapy. Additionally, Cattaneo discussed how these patient-derived T cells could be leveraged for identifying neoantigen-specific CD4 + and CD8 + T cells. Specifically, the HANSolo (HLA-Agnostic Neoantigen Screening) method allows for this by co-incubating patient-derived HLA Class I and Class II proficient immortalized B cells with the target T cells. After sequencing the patient's tumor, a custom mutanome minigene library is introduced into the B cells, and next-generation sequencing detects neoantigens by identifying B cells that are depleted upon expressing T cell-recognized epitopes [27]. Lastly, she presented how using the PDOs-T cells co-culture platform combined with mass spectrometry analysis, her team uncovered a novel in vivo mechanism of action of taxane, one of the most widely used chemotherapeutic drugs [28], showing that taxanes, such as docetaxel and paclitaxel, can activate T cells in an unconventional, TCR-independent manner, resulting in their release of cytotoxic extracellular vesicles and subsequent tumor cell death. Furthermore, by leveraging their biobank of previously generated PDO-tumor reactive T cell pairs, they demonstrated that pretreating T cells with docetaxel can enhance their TCR-dependent killing of PDOs while sparing healthy organoids. This finding suggests a promising strategy to enhance the T cell-mediated anti-tumor effects of taxane, reducing systemic toxicity and potentially avoiding cancer's taxane- resistance mechanisms.

Chiara Porta (Novara, Italy) presented recent findings on the immunological effects of Tazemetostat, an EZH2 inhibitor, in Malignant Pleural Mesothelioma (MPM) [29]. MPM is an aggressive cancer with a five-year survival rate of only about 10%, representing a critical unmet clinical need. Given its antiproliferative effects on tumor cells, Tazemetostat (EPZ-6438) is currently undergoing clinical trials (NCT02860286) for MPM. Her research team employed multicellular MPM spheroids, composed of cancer cells and monocytes, to investigate the immunological effects of Tazemetostat on 3D spheroids obtained from MPM cell lines representing the three main histological subtypes of MPM—epithelioid, biphasic, and sarcomatoid. The study highlighted that Tazemetostat promotes the expression of monocyte chemoattractants in tumor cells, which facilitates the accumulation of monocyte-derived tumor-associated macrophages (TAMs) into the MPM spheroids. Secondly, they observed that Tazemetostat impacted the functional polarization of these TAMs, pushing them towards a M2-polarized pro-tumoral phenotype, which could potentially enhance tumor resistance to treatment. These findings suggest that TAMs may play a role in mediating resistance to Tazemetostat in MPM, pointing to TAM depletion as a potential strategy to enhance the efficacy of EZH2 inhibition in this highly lethal cancer.

In summary, the session highlighted that up to today no single 3D model can meet all needs in personalized oncology. Therefore, the selection of the model best suited to address unique research or clinical questions remains critical. The session further underscored that PDOs and PDEs, in addition to providing valuable insights into foundational cancer stages—such as tumor initiation, metastasis, and disease progression, as well as on clinical applications like testing alternative therapies for relapsing or refractory cases, can also provide potential ex vivo platforms for cell therapy, able to generate patient's tailored tumor-specific T effector cells. A crucial step forward will necessarily be the standardization of protocols across clinical research institutions, both regarding the development of patient-derived models and protocols for biobanking. This standardization will improve the reliability of the results and their clinical translation.

## SESSION 3

### AI and digital pathology at the forefront of personalized diagnostics

Session 3 chaired by Hervè Fridman (Paris, France) and Paolo Dellabona (Milan, Italy) highlighted the integration of artificial intelligence (AI) and digital pathology as transformative tools poised to revolutionize the field of personalized medicine. Speakers presented advancements in machine learning algorithms capable of analyzing high-dimensional pathology data to identify diagnostic patterns and predictive biomarkers with high precision, detailing applications in optimizing patient stratification, enhancing diagnostic accuracy, and supporting decision-making in clinical workflows. Edoardo D’Imprima (Milan, Italy) presented research on the use of Correlative Light-Electron Microscopy (CLEM) for studying disease development in organoids, emphasizing the integration of light and electron microscopy to achieve nanometer-resolution imaging [30, 31]. The study utilized CLEM combined with Volume Electron Microscopy (vEM) to examine disease mechanisms in a variety of biological systems, including 2D cell cultures, organoids, animal models, and patient- derived tissues. By applying serial sample ablation and imaging techniques alongside artificial intelligence-based algorithms for automated segmentation, the research generated extensive 3D reconstructions. These models provided detailed insights into multicellular structures, allowing quantitative analysis of sub-cellular components and macromolecular interactions critical to understanding disease progression.

Mahmood Faisal (Boston, USA) presented cutting-edge data on artificial intelligence (AI) based computational pathology on whole-slide images also applied to cancers of unknown primary origin. He highlighted the need to build foundation models for pathology that could be applied in clinical practice. One of the models he presented, CONtrastive learning from Captions for Histopathology (CONCH), was developed by employing diverse sources of image-captions pairs (over 1.17 million) for task-agnostic pretraining. Moreover, a slide-level foundation model, TANGLE, integrates H&E and transcriptomics from three different organs and from two different species (Homo sapiens and Rattus norvegicus) to extract information-rich embeddings of histology whole-slide images without using explicit supervision. Finally, he showed an exceptional tool named PathChat, a vision-language generalist AI assistant for pathology with a huge impact on human pathology [32, 33].

Antonio Rosato (Padova, Italy) emphasized the importance of advanced methodologies in identifying prognostic and predictive biomarkers for patient stratification and personalized therapies [34]. Utilizing multiplex immunofluorescence (mIF) and spatial transcriptomics, he achieved high- resolution characterization of the tumor-immune microenvironment (TME). mIF enables the simultaneous detection of multiple markers while preserving spatial context, and spatial transcriptomics provides transcriptomic data within its morphological framework [35, 36]. His studies on breast cancer brain metastases and HPV-positive oropharyngeal squamous cell carcinoma (OPSCC) revealed significant immune infiltrate variations between primary tumors and metastasesIn HPV-positive OPSCC, higher immune checkpoint molecules and cytotoxic T cells correlated with better disease-free survival (DFS). These findings support incorporating immune checkpoint inhibitors into therapies tailored to specific tumor immune profiles [37].

Gianpaolo Bianchini (Milan, Italy) presented insights into spatial predictors of immunotherapy response in triple-negative breast cancer (TNBC), emphasizing the disease’s heterogeneity and the need for precision immunology. His research utilized imaging mass cytometry (IMC) to analyze 43 protein markers in both epithelial and tumor microenvironment (TME) compartments from TNBC samples in the NeoTRIP trial. The study identified immune cell phenotypes, such as proliferating CD8 + T cells (TCF1 + Ki67 +), which were associated with improved pathological complete response (pCR) following atezolizumab and chemotherapy treatment. In contrast, resistant tumors exhibited an enrichment of CD15 + tumor-associated neutrophils, suggesting immune evasion. The research highlighted the importance of baseline biomarkers, including immune cell activation and spatial distribution in the TME in predicting the efficacy of immune checkpoint blockade (ICB) therapies, underscoring the potential of IMC as a tool for precision immuno-oncology.

In the selected oral communications, Anna Tosi (Padova, Italy) presented innovative findings on spatial profiling of the TME in early triple-negative breast cancer (eTNBC), revealing mechanisms behind varied therapy responses [38, 39]. Despite a link between tumor-infiltrating lymphocytes (TILs) and prognosis, some high-TIL patients relapse after neoadjuvant chemotherapy (NACT). Using GeoMx DSP and multiplex immunofluorescence, Tosi demonstrated that non-responding high- TIL cases show reduced antigen presentation and immune activation while responding low-TIL cases exhibit enhanced antigen presentation and metabolic adaptations. In residual disease, recurrent cases had ECM-remodeling and immunosuppressive profiles, while non-recurrent cases showed robust immune activation. These insights highlight spatial profiling's role in identifying resistance biomarkers for personalized eTNBC treatment.

Alessandra Metelli (Rome, Italy) unveiled compelling evidence on the role of Tissue Factor (TF) in augmenting TGFβ signaling in cancer cells by modulating plasma membrane tension and receptor dynamics. TF, highly expressed in aggressive malignancies, was shown to influence tumor cell migration through interactions with platelet-derived TGFβ, a key driver of the epithelial-to- mesenchymal transition (EMT). Using CRISPR/Cas9-mediated TF knockout models, Metelli demonstrated that TF depletion led to epithelial-like features, including increased E-Cadherin expression, reduced Smad2/3 phosphorylation, and altered cell morphology. TF was found to regulate TGFβ receptor (TGFBR) surface expression, phosphorylation, and endocytosis by interacting with Ezrin and stabilizing its dimerization, thereby affecting plasma membrane tension. This novel mechanism links TF to the modulation of TGFβ signaling, uncovering a potential therapeutic target for antibody-based treatments in cancer patients.

Luigi Nezi (Milan, Italy) explored the long-term dynamics of gut microbiota in melanoma patients undergoing anti-PD-1 therapy, revealing microbial features associated with therapeutic response. By tracking gut microbiota over 13 months in patients from two Italian hospitals, he identified distinct microbial patterns linked to response outcomes based on PFS and RECIST 1.1 criteria. Notably, complete responders exhibited stable gut microbiota features, validated across multiple external cohorts and metagenomic studies. Nezi identified MHC-I restricted peptides from flagellin-related genes in Lachnospiraceae (FLach) as structural homologs of tumor-associated antigens, and FLach- reactive CD8 + T cells were detected in responders before therapy. These findings highlight how immune checkpoint inhibitors (ICI) therapy fosters a host-microbiota synergy, promoting immune cell function and tumor recognition in patients with favorable gut conditions. His work underscores the potential to harness or induce an ICI-conducive microbiota to enhance melanoma treatment and other solid tumor therapies.

## SESSION 4

### Tertiary lymphoid structures and microenvironment interactions as regulator of anti cancer immunity

In session 4, co-chaired by Beatrice Belmonte (Palermo, Italy) and Paola Nisticò (Rome, Italy) the speakers emphasized the complex interactions between B and T cells and their role in anti-cancer immunity and immunotherapy response. They highlighted emerging research on Tertiary Lymphoid Structures (TLS) composition and localization and as pivotal players in the Tumor Immune MicroEnvironment (TIME) to identify novel biomarkers and likely targets for effective immunotherapeutic approaches.

Focusing on the heterogeneity and plasticity of T cells in the TIME, Massimiliano Pagani (Milan, Italy) presented data on the epigenetic landscape of TILs by analyzing genome-wide chromatin accessibility using ATAC-seq in colorectal cancer and non-small cell lung cancer patients. The findings highlighted distinct chromatin accessibility patterns in TILs compared to normal lymphocytes, particularly in tumor-infiltrating CD4 + regulatory T cells (TI-Tregs). By examining chromatin regions and transcription factor occupancy, preferentially accessible regulatory regions in TI-Tregs were identified, and BATF was found as a key transcription factor. These results provide new insights into the molecular profile of tumor-infiltrating Tregs through their unique chromatin accessibility patterns.

Tullia Bruno (Pittsburgh, USA) presented an insightful analysis of B cells by spatial transcriptomics in High-grade serous ovarian cancer (HGSOC), revealing differences across different anatomical sites. She reported that B cells are more active when aggregated in lymphoid structures, although heterogeneous depending on anatomical local metastatic sites, such as fallopian tube (FT) or omentum (OM), and highlighted the different functional activity of T and B cells when localized inside or outside TLS. Focusing on stromal cells as critical regulators of immune infiltration and function, data shown revealed that Cancer-Associated Mesenchymal Stem Cells (CA-MSCs) exert an inhibitor role on TLS, whereas normal MSCs express genes associated with TLS-related chemokines. She identified a 20-gene transcriptional signature as a prognostic factor, mainly in HGSOC young patients. The question of how immune-related targets may affect TLS formation and function still remains to be explored. Catherine Sautes-Fridman (Paris, France) explored the intricate relationship between tumor cells and TLS shaping, focusing on clear cell renal carcinoma (ccRCC) and liposarcoma. In ccRCC, high expression of a GABAergic signature in TLS-adjacent tumor cells correlated with poorer outcomes and immunotherapy non-responsiveness, suggesting a suppressive role of the GABA pathway on anti-tumor immunity. Non-responder patients displayed TLS with low cytotoxic T cell activity, abundant M2 macrophages, immature dendritic cells, and naïve B cells, coupled with diminished antigen presentation, IgA elevation, and reduced IgG. In liposarcoma, dedifferentiated high-grade regions exhibited immune exclusion with reduced TLS and immune cell infiltration, highlighting immune desertification. These findings underscore the pivotal role of TLS and spatial immune dynamics in shaping the TME and modulating therapeutic responses.

Anna Dimberg (Uppsala, Sweden) proposed an innovative strategy to address immunotherapy resistance in glioblastoma, a highly aggressive brain tumor with an immune-excluded microenvironment. She hypothesized that by changing tumor vessels into high endothelial venules (HEV), an anti-tumor immunity enabling T cell infiltration and TLS formation may be induced. Treatment of glioma-bearing mice with an adeno-associated virus (AAV) targeted to brain endothelial cells and coding for the cytokine LIGHT/TNFSF14 reduced tumor growth, and a proportion of the mice completely cleared the tumors. This was associated with the induction of tumor-associated HEVs and the formation of TLS, as well as an accumulation of stem-like TCF1 + PD1 + CD8 T cells. Tumor clearance was associated with effector/memory anti-tumor T cell responses. They are currently developing new AAV variants with tropism for human tumor vessels to enable translation of this finding.

In the oral communication session, Nicla Porciello (Rome, Italy) presented novel findings on Non- Small Cell Lung Cancer (NSCLC), underscoring the significance of TLS as biomarkers associated with improved outcomes in patients receiving Immune Checkpoint Blockade (ICB) therapy. By exploiting different spatial transcriptomics approaches, this study emphasizes how the cellular composition and spatial organization within the TIME play a pivotal role in shaping anti-tumor immune responses. A key predictive signature comprising hMENA, fibronectin, and Lymphotoxin β Receptor (LTβR) was identified in TLS-enriched TIME and correlated with favorable ICB responses [40]. She noted that notable variations in the distribution of T and B cells across patients occur, with activated and memory B cells particularly enriched within TLS of patients responding to therapy. She put the spotlight on stromal cells and, in particular different Cancer-Associated Fibroblasts subtypes able to influence TLS maturation and function. These insights suggest that specific biomarkers could significantly enhance patient stratification and the effectiveness of ICB therapies in NSCLC.

In the Selected Oral Communications Session, Paola Cappello (Torino, Italy) presented a compelling study investigating the impact of the hemochromatosis H63D polymorphism (HFE), a genetic variant associated with hereditary hemochromatosis, on the progression of pancreatic ductal adenocarcinoma (PDAC) and its influence on the antitumor immune response. Tumors harboring the H63D mutation demonstrate increased aggressiveness, a heightened propensity for metastasis, and frequent neuronal invasion within the pancreas. Furthermore, the H63D mutation is associated with a shift towards a type-II immune response, characterized by elevated antibody production. While this immune response may partially counter tumor growth, it concurrently appears to facilitate tumor progression through immune suppression. These findings suggest that the H63D polymorphism could serve as a novel biomarker for PDAC prognosis, potentially guiding patient stratification and informing tailored treatment strategies.

Valeria Cancila (Palermo, Italy) explored T-cell exclusion in lymphomas using the germinal center (GC) as a model. The GC, divided into the dark zone (DZ) for B-cell hypermutation and the light zone (LZ) for immune refinement, served to study T-cell distribution and immune evasion. Spatial transcriptomics revealed that the DZ restricts T-cell infiltration via active DNA damage response (DDR) pathways, chromatin compaction, and suppressed inflammation, a profile linked to aggressive diffuse large B-cell lymphomas (DLBCLs). ATR kinase emerged as a key regulator of this immune- repellent environment. ATR inhibition (ATRi) reprogrammed the DZ, reducing immune suppression and enabling T-cell migration toward lymphoma cells. These findings propose ATR inhibition as a novel strategy to enhance immunotherapy in immune-excluded lymphomas.

## SESSION 5 – Vaccines and beyond

Immune checkpoint inhibitors have generated a paradigm shift in anticancer treatment. Anticancer vaccines most recently developed, taking into account stringent antigen selection and more effective delivery systems, represent promising clinical implications.

Session 5 of the NIBIT congress, co-chaired by Anna Mondino (Milan, Italy) and Sheila Spada (Rome, Italy), was indeed dedicated to the most recent advancements in the field of cancer vaccines and DC-targeted therapies. Presentations spanned the genetic modulation of tumor antigens and tumor microenvironment in preclinical mouse and dog models and the employment of peptide-based vaccines in cancer patients.

Maria Rescigno (Milan, Italy) introduced the concept of disease-specific universal vaccines based on immunogenic peptides released by cancer cells and their use against metastatic melanoma, sarcoma, and osteosarcoma. She also discussed the consequence of Salmonella infection as a strategy to promote the release of immunogenic peptides, able to trigger protective anti-tumor immunity. Rescigno presented novel data showing that the secretome of Salmonella-treated tumor cells achieved CD8 T cell-dependent anti-tumor effects in sarcoma and melanoma-bearing dogs. A clinical study to test the vaccine in combination with an anti-PD1 antibody is now being planned for metastatic melanoma patients.

Federica Benvenuti (Trieste, Italy) highlighted the role of type 1 dendritic cells (cDC1) in regulating neoantigen-specific antitumoral responses in lung cancer. She presented a mouse model suitable to address the function of cDC1 in specific tissue. More in detail, by the genetic deletion of mismatch repair regulator Mlh1 gene, they created a murine hypermutated model of antigenic and yet poorly immunogenic KRAS/TP53 mutant (KP) NSCLC (KP^neo^). They exploited such a model to study.

MHC-I neoepitopes-specific CD8 T cell responses in response to spontaneous tumors and upon DC therapy (Flt3L + αCD40). Data indicated that vaccination of tumor-bearing mice with DC pulsed triggered antigen-specific CD8 T cell expansion and a partial control of tumor growth. Of note, vaccination instructed the reshaping of the lung immune contexture, favoring the expansion of cDC1 with immunostimulatory properties and limiting exhausted CD8 T cells accumulation (PMID: 38480738). cDC1 accumulation also favored antigen cross-presentation within the tumor microenvironment and the upregulation of interferon signaling.

Roberto Chiarle (Torino, Italy) discussed the possibility of exploiting the anaplastic lymphoma kinase (ALK) as a novel target for CAR-based T-cell therapy and for peptide-based vaccination in pediatric neuroblastoma. He showed that human ALK-CART cells efficiently recognize and control tumor growth with high ALK expression while showing modest reactivity against ALK-low metastatic neuroblastoma cells. Interestingly, treating mice with Lorlatinib, a third-generation of ALK-targeted small molecule inhibitor, enhanced ALK-CART reactivity to ALK-low targets. A first- in-human anti-ALK immunotherapy, combining ALK-CART in combination with Lorlatinib, has been planned [41]. Chiarle also discussed the results of a phase I trial exploiting an ALK vaccine to treat patients with NSCLC. He showed that the ALK-peptide vaccine elicited strong and specific immune responses, which could be further augmented by the combination with ALK TKI and ICI [42].

Oral selected Communications were presented by Riccardo, Barutello and Shallak.

Federica Riccardo (Torino, Italy) showed preliminary data related to the targeting of the chondroitin sulphate proteoglycan (CSPG)4 in malignant melanoma models. Specifically, she demonstrated that a DNA vaccine encompassing either human (Hu) and dog (Do) CSPG4 (HuDo-CSPG4) overruled the host’s unresponsiveness, promoting immunity. A prospective veterinary trial on client-owned dogs with surgically resected CSPG4- positive oral malignant melanoma (MM) showed that anti- CSPG4 vaccination was well tolerated and highly immunogenic. In addition, vaccination significantly extended dog survival compared to controls. Riccardo suggested that data provide the rationale for the combination of the HuDo-CSPG4 vaccine with available anticancer therapies in MM patients or other CSPG4-expressing malignancies [43].

Giuseppina Barutello (Torino, Italy) presented preliminary data on the prometastatic role of teneurin-4 (TENM4) in both in vitro and in vivo triple-negative breast cancer (TNBC) models [44] TENM4-deficient TNBC cells were less prone to form tumor spheres-, had less clonogenicity and less ability to migrate and invade the lungs. High-TENM4 TNBC harbored increased expression of MMP2, ADAM12, and significant decreased expression of TIMP2 compared to low-TENM4- expressing primary tumors. Of note, TENM4 gene-based vaccination is safe, and it is able to induce an anti-TENM4 cellular and humoral response, suggesting that TENM4 could be considered a novel target for developing future cancer vaccines in TNBC.

Mariam Shallak (Varese, Italy) presented some preliminary findings regarding MHC-II restricted peptide vaccination in the murine glioblastoma model. She showed the stable expression of the MHC class II transactivator (*CIITA*) caused de novo expression of MHC-II molecules in glioblastoma cells, and this was required for anti-tumor effects, suggesting that CIITA-induced MHC-II expression could reveal a suitable strategy to unveil novel MHC-II restricted tumor-specific peptides suitable for vaccine development [45].

In summary, findings from session 5 suggest that strategies able to promote antigen expression and presentation in favorable anticancer immune environments favor the establishment of protective anticancer immunity initiated by cancer vaccines.

## SESSION 6

### Clinical Perspectives: Translational, Platform, Basket and Neoadjuvant Trials

Session 6, chaired by Pier Francesco Ferrucci (Milan, Italy) and Claudio Tripodo (Milan, Italy), focused on novelties in clinical cancer immunotherapy, perspectives and applications, showing how translational tools are driving precision oncology into reality.

The first speaker of the session was Christian Blank (Amsterdam, Netherlands) pioneered neoadjuvant immune checkpoint inhibition in melanoma following the idea that immunotherapy is most effective when the tumor is still present with all its immuno-variety. His group found unparalleled high pathologic responses in this setting of locally advanced melanoma, which were associated with almost no recurrences. In particular, PRADO and NADINE are practice changing trials and showed that Neoadjuvant immunotherapy is the new standard of care for macroscopic stage III melanoma. In fact, said Prof. Blank, patients receiving neoadjuvant ipilimumab + nivolumab could achieve up to 65% of Major Pathological Response (MPR). When an MPR is reached in their indexed lymph node (ILN), likely, tumor lymph nodal dissection (TLND) and even adjuvant therapy can be omitted. On the other hand, non-MPR patients are clearly benefiting from adjuvant treatment.

Neoadjuvant therapies also provide a translational research opportunity to explore gene expression signatures associated with response and to understand molecular mechanisms of resistance, defining the extent of immunotherapy needed. In fact, it seems that non-responders have an impaired systemic immune activatability rather than an immune-suppressed tumor microenvironment. In particular, escape after response seems to be due to’cooling down’ of the immune response rather than genetic changes mediating immune escape. In that way, prof. Blank presented data on how to develop personalized neoadjuvant immunotherapy by dissecting toxicity from the response and allowing escalations towards baseline- and on-treatment signature-driven mono, double triple, and quadruple therapies [46–49].

The second speaker of the session was Ciro Celsa (Palermo, Italy) who focused his presentation on the rapidly increased number of effective treatments for advanced hepatocellular Carcinoma (HCC), leading to the development of several potential sequential or combination strategies of systemic therapies. However, said Celsa, the best treatment strategy for these patients remains elusive.

In the first-line setting, the combination of atezolizumab plus bevacizumab resulted in more effective than sorafenib in improving survival and quality of life, becoming the new standard of care for the upfront treatment of advanced HCC. However, its safety and cost-effectiveness remain to be established in a real-world setting of patients with cirrhosis and portal hypertension.

Although several second-line systemic therapies are now available, including both multikinase inhibitors and immune checkpoint inhibitors, randomized controlled trials evaluating sequential strategies are lacking and difficult to perform, so some controversial issues remain open. For example, it is unclear if the optimal treatment strategy is the sequence, or the combination and which agents should be employed in order to maximize the net health benefit for these patients.

In advanced, intermediate, and early-stage HCC, Celsa explained that immune ICI-based combinations are the standard of care, but biomarkers to predict response are not available in clinical.

practice. Transcatheter arterial chemoembolization (TACE) plus ICIs improve progression-free survival in the intermediate stage, but overall survival benefit remains to be assessed. On the other hand, adjuvant treatment remains an unmet need, while neoadjuvant s needs further evaluation in phase III trials.

Waiting for results from ongoing trials and real-world evidence arising from field-practice studies, simulation models could inform clinical decision-making and can be used to assess the net health benefit of the available systemic treatments [50–52].

Giuseppe Curigliano (ESMO President Elect, Milan, Italy) focused his presentation on the personalized treatment of metastatic breast cancer (MBC), which has been enriched in recent years with targeted therapies, antibody–drug conjugates (ADCs), and immunotherapies.

Immunotherapy is available for PD-L1 positive tumors and has already changed the therapeutic algorithm of triple-negative breast cancer patients (TNBC). PARP inhibitors are very effective in BRCA-mutated cancers, while significant advancements have been made in the metastatic setting using antibody- drug conjugates (ADCs) like Sacituzumab Govitecan.

Sacituzumab Govitecan is now the standard of care option for patients with metastatic triple-negative breast cancer in the second line and beyond settings (or early relapsers). It is a humanized monoclonal antibody (hRS7 IgG1κ) that recognizes Trop-2. Instead, the small molecule SN-38 is an inhibitor of topoisomerase I, which is linked with a covalent bond to the antibody via a hydrolyzable linker. However, due to the development of secondary resistance, patients often experience disease progression, and no clear evidence is available on ADCs sequencing strategies and combination approaches in the treatment of MBC.

Concerning HER2-positive MBC, current evidence on the optimal ADC-sequencing is primarily about trastuzumab deruxtecan (T-DXd), which demonstrated therapeutic value when used post- Trastuzumab emtansine (T-DM1), the first approved ADC. Conversely, data are limited about the reverse sequence. Similarly, said Curigliano, in HER2-negative MBC, recent studies evaluated the sequential use of Sacituzumab Govitecan and T-DXd, which was associated with poor responses.

Retrospective analyses have not demonstrated an optimal sequencing strategy for ADCs, and it is still very unclear whether switching the payload or targeting a different antigen may represent the best approach. Combinations may better overcome ADC resistance: interesting data associating immunotherapy or tyrosine kinase inhibitors to ADCs appear promising, albeit data are still immature. In particular, data on the use of ADCs in residual disease and in earlier-line settings, as well as ICI and ADC combinations, are awaited.

In summary, Curigliano explained that in MBC, ADCs have expanded treatment options, but their sequential use requires further study. Evidence suggests that sequencing ADCs with similar payloads is ineffective, though current data are inconclusive. More research is needed to optimize treatment strategies, including potential combination therapies [53–56].

The selected abstracts for oral communications of this session were presented by Corsale (Palermo, Italy), Cogrossi and Damiano (Milan, Italy).

Anna Maria Corsale presented her data on the interplay between clonal plasma cells and the bone marrow (BM) microenvironment, which plays a crucial role in the development of multiple myeloma (MM). She said that understanding this relationship is crucial for identifying and effectively managing patients at a high risk of neoplastic progression, ultimately improving clinical outcomes. γδ T cells, functioning as a link between innate and adaptive immune systems, contribute to immune responses during cancer progression. However, their role in MM and its early phases, such as monoclonal gammopathy of undetermined significance (MGUS) or smoldering MM (SMM), remains unclear. In particular, γδ T cells acquired a dysfunctional phenotype and impacted patient prognosis. Circulating γδ T cells mimic the bone marrow microenvironment and the expression of TIM3 on γδ

T cells may represent a potential biomarkers for disease progression and a therapeutic target in MM. In conclusion, Corsale data sustain the role of BM γδ T cells during MM progression by acquiring a dysfunctional phenotype, which significantly impacts patient prognosis. Accordingly, circulating γδ T cells may serve as a potentially less invasive biomarker for follow-up and prognostication of patients affected by monoclonal gammopathies [57]. For her abstract, Corsale received the Grazia Focacci Foundation award.

On the same track, Laura Lucia Cogrossi explained how poor diet quality, elevated body mass index (BMI), insulin resistance, microbiome dysbiosis, inflammation as well as immune dysfunction have all been implicated in the progression from MGUS and SMM to MM. Plant-based diets have been associated with reduced risk of MGUS and MM in epidemiological studies.

Cogrossi presented data on the first clinical trial and *in vivo* study to show that a high fiber plant-based diet intervention may delay progression from MGUS/SMM to MM. In this trial, patients reported improvement in metabolic and obesity-related parameters as well as benefits in overall quality of life. Dietary fiber consumption improved immune response and expanded short-chain fatty acid-producing microbiota with anti-inflammatory and antitumoral function.

In conclusion, Cogrossi said that this area of investigation has the highest chance to delineate microbiota-related and pathobiology-based parameters for patient risk stratification [58].

Giuseppe Damiano presentation focused on metabolism and its influence on both tumor and immune cells. Several reports have recently shown that the pharmacologic and genetic manipulation of metabolic pathways restores antitumor immune responses by activating or suppressing distinct cellular elements in the tumor microenvironment (TME). In particular, Simvastatin can lead to a synergistic effect with anti-PD1 improving antigen presentation on intratumoral dendritic cells.

Damiano and colleagues, in order to investigate the efficacy and biological mechanisms of simvastatin plus anti-PD1, analyzed PFS and OS from a cohort of metastatic melanoma patients and performed in vivo experiments to compare the effect of simvastatin, anti-PD1, or combination of simvastatin plus anti-PD1 in C57BL/6 mice bearing the B16F1 melanoma. In the patient’s melanoma cohort, 2-year PFS was 50% vs. 10% (p=0.01), 2-year OS 75% vs. 50% (p=0.03), and the median OS was 24 months vs. not reached in favor of statin-treated patients. Moreover, pre-clinical experiments in mice showed that the combinatorial treatment (simvastatin plus anti-PD1 mAb) was capable of slowing down tumor growth.

In conclusion, Damiano, who received the “Melagioco” Award for melanoma, observed an increased PFS and OS in metastatic melanoma patients taking statins and treated with checkpoint inhibitors and replicated this effect on melanoma-bearing mice, in which he also observed intratumoral DCs expressing higher levels of activation markers. In vitro, he showed the upregulation of activation markers as well as increased p38 phosphorylation and pro-inflammatory cytokine expression in simvastatin-treated DCs, saying that these data might explain the improved efficacy of immunotherapy in melanoma patients taking statins [59].

## Conclusion

Technology is advancing rapidly, offering critical insights into the interactions between cancer cells, immune cells, and therapeutic agents. These advancements open new avenues for studies that could enable us to match the right patient with the optimal target, setting, timing, and dosing of treatments. We stand on the brink of a transformative era in clinical therapeutics. However, challenges persist in designing informative studies and navigating the complexities of analyzing and interpreting vast datasets. To address these hurdles, artificial intelligence is being leveraged, unlocking a pivotal opportunity to reshape the therapeutic landscape.

## Data Availability

No datasets were generated or analysed during the current study.
